# Supplementary material for: Maternity waiting homes utilization and associated factors among women who gave birth in the last one year in rural settings of Basona Worena District, Ethiopia: A cross sectional study
Source: PLoS One. 2025 Oct 6;20(10):e0331624. doi: 10.1371/journal.pone.0331624 (PMC12500156; doi:10.1371/journal.pone.0331624)
Supplement: Attachment — Submitted filename: Response to reviewers.docx [file pone.0331624.s002.docx]

**Letter of response**

**To all honorable reviewers and editors**

Dear Reviewers and Editors, We are very appreciative for your pertinent comments, questions and recommendations. Following this, we have provided our responses point by point as presented below.

**Authors Responses**

1. Is the manuscript technically sound, and do the data support the conclusions?

The manuscript must describe a technically sound piece of scientific research with data that supports the conclusions. Experiments must have been conducted rigorously, with appropriate controls, replication, and sample sizes. The conclusions must be drawn appropriately based on the data presented.

Reviewer #1: Partly

Reviewer #2: Yes

**Answer**

**We used a cross-sectional study design, and the findings are supported by statistical analysis and scientific description. These findings are important for maternal health and sound science for responsible bodies.**

2. Has the statistical analysis been performed appropriately and rigorously?

Reviewer #1: I Don't Know

Reviewer #2: Yes

**Answer**

**Our study's outcome variable was binary (yes/no), and binary-logistic regression was the statistical model that fit it. The model's fitness (p=0.601) allowed it to function as intended. Thus, our approach to analysis is appropriate and thorough.**

3. Have the authors made all data underlying the findings in their manuscript fully available?

Reviewer #1: Yes

Reviewer #2: Yes

**Answer: NA**

4. Is the manuscript presented in an intelligible fashion and written in Standard English?

Reviewer #1: No

Reviewer #2: Yes

**Answer**

**We are accepted and amend it.**

5. PLOS authors have the option to publish the peer review history of their article (what does this mean?). If published, this will include your full peer review and any attached files. If you choose “no”, your identity will remain anonymous but your review may still be made public. Do you want your identity to be public for this peer review? For information about this choice, including consent withdrawal, please see our Privacy Policy.

Reviewer #1: No

Reviewer #2: No

**Answer: NA**
