# Supplementary material for: Maternity waiting homes utilization and associated factors among women who gave birth in the last one year in rural settings of Basona Worena District, Ethiopia: A cross sectional study
Source: PLoS One. 2025 Oct 6;20(10):e0331624. doi: 10.1371/journal.pone.0331624 (PMC12500156; doi:10.1371/journal.pone.0331624)
Supplement: Attachment — Submitted filename: Response_to_reviewers_auresp_2.docx [file pone.0331624.s003.docx]

**Letter of response**

**To all honorable reviewers and editors**

Dear Reviewers and Editors, We are very appreciative for your pertinent comments, questions and recommendations. Following this, we have provided our responses point by point as presented below.

**Authors Responses**

**Abstract**
“However, little is known in Ethiopia on the utilization of maternity waiting home among actual mothers who gave birth in rural settings and their involvement of the service utilization is not well explored.” revise this sentence
“In Ethiopia, the utilization of maternal waiting homes and its associated factors among women who gave birth in rural setting were not clearly described.” redundancy of the very earliest sentence.
Please, correct the tense error from the objective
“(>=5)”would be written in clear term.
“The overall maternity waiting home utilization was 56.7% which relatively low compare to previous studies” grammatical error.

**Answer =**

**Accepted and we amend it**.
**Introduction**
This section must be revised. It lacks consistency and coherence.
“Most of the scholars focused on estimating the intention and knowledge of mothers to utilize MWHs for their current delivery” cite this.
“However, little is known in Ethiopia on the utilization of MWHs among actual mothers who gave birth in rural settings and their involvement of the service utilization is not well explored. In Ethiopia, the utilization of maternal waiting homes and its associated factors among women who gave birth in rural setting were not clearly described. However, no study was found during the literature review period that had been shown in Basona worena district.” revise these sentences too. Pay attention.

**Answer =**

**Accepted and we amend it**.

**Methods and materials**
Please, use universally acceptable expressions, where all scientists can understand. For instance, what is 2015 E.C.?
**Variables (Figure 2)?**
The authors would cite the source of their tool and append their Cronbach's Alpha test value

**Answer =**

**Accepted and we amend it**.
**Results:**
The percentages of observations of some variables are not correct.
Replace “Reproductive health Characteristics of participants” by Obstetrics characteristics of participants. Because reproductive health is a broad term or not limited to the characteristics you mentioned in this study.
The independent variables depicted in the variable section and the result section are inconsistent.
Replace “Multivariate logistic regression” by factors associated with MHW utilization among participants.
Line 288-292 is the redundancy of data analysis method. The result section should only include results.
“Those mothers whose age category was aged between 26-30 years old were 0.22 times more likely to utilized MWH than those women whose age category was 36 and above (AOR=0.22,95% CI:0.08,0.65).” Check this interpretation
Put the row percentages of yes and no observation of MWH utilization in table 5

**Answer =**

**Accepted and we amend it**.
**Discussion**
Note that family size and parity are different variables.
Studies limitations would be mentioned under this section and remove the strength you have mentioned. A sampling technique that was based on your study’s nature and design should not mention as study’s strength.

**Answer =**

**Accepted and we amend it**.
**Conclusion**
The conclusion seems like results. Not appropriately written
some the recommendations are not based on your findings

**Answer =**

**Accepted and we amend it**.
**Ethical approval**
How could you take a written informed consent form those 33.5% your study participants who were unable to read and write? This should be clarified.

**Answer =**

**This is important point so we used both written and oral consent methods.**
**General suggestions**
Figures are not numbered correctly.
Omit meaningless arrows from the figure that illustrated sampling procedure
the manuscript has serious grammar and punctuation errors. So the authors must use language edition services.
